# Resident Proficiency in Assessing Medical Decision-Making Capacity: A Needs Assessment and Educational Intervention

**DOI:** 10.7759/cureus.114190

**Published:** 2026-08-09

**Authors:** Samuel D Cartwright, Tyler Indranoi, Jacob S Snipp, Aamir Shaikh, Caleb W Brown, Venkata Vedantam

**Affiliations:** 1 Internal Medicine, Quillen College of Medicine, Johnson City, USA; 2 Psychiatry, Quillen College of Medicine, Johnson City, USA; 3 Surgery, Quillen College of Medicine, Johnson City, USA

**Keywords:** graduate medical education, medical decision-making capacity, medical ethics, quality improvement, resident education

## Abstract

Background

Medical decision-making capacity is fundamental to informed consent, patient autonomy, and ethical care, yet incapacity is frequently underrecognized. Despite encountering patients requiring capacity assessments, residents often receive limited formal training.

Objective

The objectives of this study are to identify gaps in resident knowledge, confidence, and perceived barriers to capacity assessment and evaluate the impact of a targeted educational intervention.

Methods

This IRB-approved quality improvement study (2025-2026) included residents from two family medicine and one internal medicine residency program (83 eligible participants). A voluntary pre-survey assessed resident experience and educational needs. Primary measures include self-reported knowledge, confidence, understanding of Appelbaum's criteria, distinction between capacity and competence, documentation skills, and legal/ethical considerations using 5-point Likert scales. Secondary measures included prior training, frequency of capacity assessments, perceived barriers, and educational preferences. Survey findings were utilized to create a targeted educational intervention. This was designed to be a one-hour interactive, case-based didactic addressing Appelbaum's four components of capacity, capacity versus competence, documentation, and legal/ethical considerations. A voluntary post-survey was administered to compare primary measures to pre-survey results. The population size for the educational intervention and post-survey had a maximum of 63 participants due to one family medicine program withdrawing. Independent t-tests compared pre- and post-intervention responses.

Results

Seventy-one residents (86% response rate) completed the pre-survey and 40 (63% response rate) completed the post-survey. Although 58% (n=41) reported performing capacity assessments weekly, no prior training was reported by 63% of PGY1s (n=15), 46% of PGY2s (n=12), and 19% of PGY3s (n=4). Common barriers included legal/ethical aspects at 70% (n=50) and time constraints at 62% (n=44). Mean scores improved significantly after the intervention for understanding of capacity components (3.44-4.55), capacity versus competence (3.15-4.60), documentation (3.00-4.68), legal/ethical considerations (2.99-4.70), confidence (3.28-4.62), and knowledge (2.17-4.78) (all *p*<0.001).

Conclusions

Residents frequently perform capacity assessments despite limited formal training. An educational intervention significantly improved self-reported knowledge and confidence, supporting incorporation of structured capacity assessment curricula into graduate medical education.

## Introduction

Medical decision-making capacity is essential to informed consent, patient autonomy, surrogate decision-making, and ethically appropriate care. Incapacity is common but frequently underrecognized. Statistics show that 26% of medicine inpatients lacked decision-making capacity, yet clinicians recognized incapacity in only 42% of affected patients [1,2]. A previous study found that factors limiting a physician’s ability to assess capacity primarily stemmed from a lack of knowledge and formal learning opportunities. The same study additionally discovered few educational opportunities were provided throughout residency [3]. Others have gauged the misconceptions regarding capacity assessment. Specifically, Ganzini et al. found that physicians commonly equate cognitive impairment with incapacity and assume capacity is an all-or-none phenomenon [4]. These studies demonstrate a clear need for improvement in medical decision-making capacity education. Although prior studies regarding educational interventions have demonstrated improvements in resident confidence and decision-making skills, few have simultaneously evaluated baseline educational needs and resident-perceived barriers before designing the intervention [5]. 

This quality improvement project aims to further identify and quantify gaps in resident knowledge, confidence, and perceived barriers in assessing medical decision-making capacity and then address those gaps through a targeted educational intervention. We hypothesized that residents would report a low level of self-confidence and a poor knowledge base of capacity assessment guidelines, similar to the results of current research. Further, the hypothesis included that an intervention based on the pre-survey needs assessment would improve resident knowledge of capacity and how to assess it. 

## Materials and methods

This institutional review board (IRB)-approved uncontrolled pre-post quality-improvement study conducted from 2025 to 2026 included residents from three residency programs at a single academic institution: two family medicine programs and one internal medicine program. The resident programs staff multiple hospitals and primary care clinics within Central Appalachia. These include multiple community hospitals, a Veterans Affairs hospital, and one Level One Trauma Center. Psychiatric consultation services are only physically present at one medical institution but are available for tele-consult at all facilities. None of the three resident programs have a formal curriculum for medical-decision-making capacity assessment. 

The study population consisted of a potential maximum of 83 eligible residents. Data were collected using anonymous, voluntary electronic surveys (see the Appendices) administered through the platform Google Forms. The survey was designed in such a way that each question was required for submission, ensuring no incomplete data was submitted. The study was split into two phases: a pre- and post-survey. The in-house designed pre-survey assessed resident demographics, prior training, and frequency of capacity assessments with direct, multiple-choice questions. It then addressed perceived challenges, patient factors, and preferred educational formats with multiple-response questions. Lastly, a baseline of each resident’s self-reported confidence/knowledge was assessed using a 5-point Likert scale regarding the four components of Appelbaum’s criteria [6], capacity vs. competence, documentation skills, ethical vs. legal concerns, and baseline knowledge improvement. The pre-survey results were utilized to create an educational intervention based on feedback. An hour-long, in-person, interactive, case-based didactic was designed. The presentation and materials were designed by an in-house psychiatry attending and PGY-3 psychiatry resident. Education resources were accessed at no cost, and no financial compensation was provided. This was presented six months after the pre-survey to a potential population of 63 instead of 83 as one of the family medicine residencies elected not to participate. The follow-up, anonymous, voluntary post-survey was administered after the educational intervention in the same fashion. It assessed the metrics of the aforementioned knowledge/confidence levels with the same 5-point Likert scale. Data analysis was performed using RStudio to calculate independent t-tests, mean scores, p-values, and confidence intervals (CIs) of the pre- and post-survey results (with primary measures being self-reported confidence and knowledge around the four components of Appelbaum’s criteria, capacity vs. competence, documentation skills, ethical vs. legal concerns, and baseline knowledge improvement). 

## Results

The pre-survey had 71 responses, representing an overall response rate of 86%. This consisted of 57.7% internal medicine (n=41) and 42.3% family medicine residents (n=30). Table 1 represents the demographics of the survey population. 

**Table 1:** Pre-survey Study Population PGY1: Post-Grad Year 1 Residents; PGY2: Post-Grad Year 2 Residents; PGY3: Post-Grad Year 3 Residents; IM: Internal Medicine Residency; FM: Family Medicine Residency

Program/Year	Number of Residents	No Training	Percent without Training
Total Overall PGY1	24	15	63%
Total Overall PGY2	26	12	46%
Total Overall PGY3	21	4	19%
Total Overall Population	71	31	44%
IM PGY1	14	7	50%
IM PGY2	14	6	43%
IM PGY3	13	2	15%
Total IM Population	41	15	37%
FM PGY1	10	8	80%
FM PGY2	12	6	50%
FM PGY3	8	2	25%
Total FM Population	30	16	53%

Prior training among residents was limited with 63% of PGY1s (n=15), 46% of PGY2s (n=12), and 19% of PGY3s (n=4) reporting no prior training on capacity assessment. Capacity assessments were reportedly performed daily by 23% (n=16), weekly by 35% (n=25), and monthly by 21% (n=15) of residents. Another 21% (n=15) stated they never or rarely performed capacity assessments. The most common challenges residents faced regarding capacity included legal/ethical aspects at 70% (n=50) and time constraints at 62% (n=44). Other factors included communication at 46% (n=33) and lack of supervision at 41% (n=29). The most common patient factors encountered included psychiatric disorders at 76% (n=54), acute medical illness at 70% (n=50), and cognitive impairment at 66% (n=47). Other patient factors included language barriers at 37% (n=26), cultural differences at 24% (n=17), and intoxication at 6% (n=4). Mean levels for the quantitative confidence/knowledge questions are represented in Table 2. Finally, for the educational intervention, 48% (n=34) residents requested a didactic session, 49% (n=35) requested case based, and 4% (n=3) requested online modules. Topics requested for the didactic were documentation at 66% (n=47), followed by legal standards at 62% (n=44), handling disagreements at 48% (n=34), and communication skills at 42% (n=30). 

**Table 2:** Independent t-test Results

Component Assessed	Pre-Survey Mean	Post-Survey Mean	Mean Difference (95% CI)	p-value
Appelbaum's Criteria	3.44	4.55	+1.11 (0.77-1.45)	<0.001
Capacity vs. Competence	3.15	4.60	+1.45 (1.10-1.79)	<0.001
Documentation Skills	3.00	4.68	+1.68 (1.34-2.01)	<0.001
Ethical vs. Legal Issues	2.99	4.70	+1.71 (1.40-2.03)	<0.001
Confidence Level	3.28	4.62	+1.34 (1.02-1.67)	<0.001
Knowledge Level	2.17	4.78	+2.61 (2.41-2.95)	<0.001

An hour-long, in-person, didactic/case-based intervention was designed by an in-house psychiatry attending and PGY-3 psychiatry resident, which focused on educating residents on capacity assessment using a case from Fitzgerald Health Education Associates [7]. Following the case, a lecture regarding the four pillars of Appelbaum’s criteria (understanding, appreciation, reasoning, and expression), indications to perform a capacity assessment, clinical assessment tools, Tennessee state medical laws (TCA § 68-11-1701) [8], and documentation skills was given. 

Following the didactic session, 40 residents completed the post-survey, representing a 63% response rate. Mean scores, 95% CI, and p-values are reported in Table 2. 

**Figure 1:**
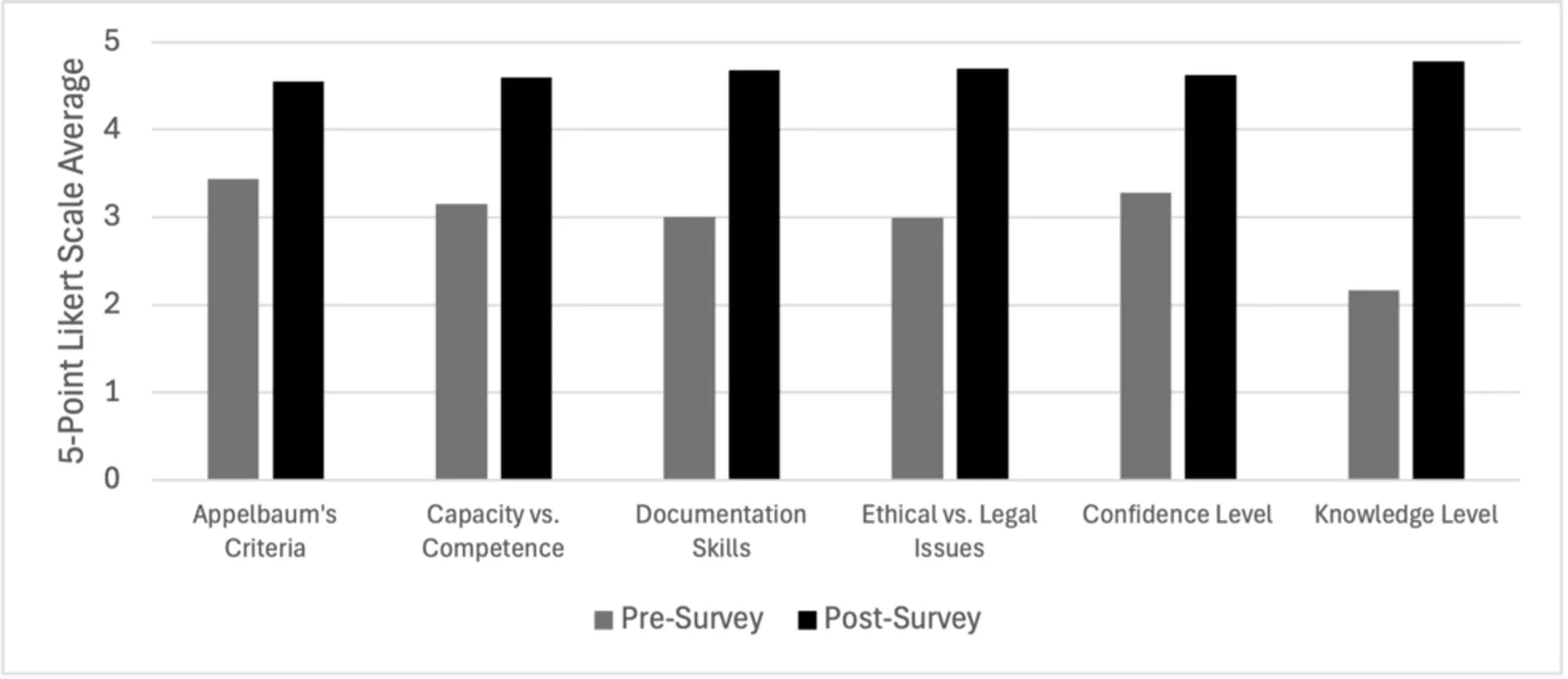
Pre- and Post-survey Mean Comparison

Mean scores improved across all measured domains: understanding the four components of capacity increased from 3.44 to 4.55, capacity versus competence from 3.15 to 4.60, documentation from 3.00 to 4.68, ethical versus legal considerations from 2.99 to 4.70, confidence from 3.28 to 4.62, and knowledge from 2.17 to 4.78. All comparisons were statistically significant with p < 0.001. The averages of these scores are compared in Figure 1.

## Discussion

This quality improvement study identified significant gaps in resident education regarding medical decision-making capacity despite frequent exposure to capacity assessments in clinical practice. More than half of residents reported performing capacity assessments at least weekly, yet nearly half of all respondents reported no prior formal training. This discrepancy highlights an important educational gap, as residents are routinely expected to make decisions regarding patient autonomy, informed consent, and surrogate decision-making without standardized instruction. 

The pre-survey findings demonstrated that residents requested information regarding documentation and legal standards. These findings are consistent with previous literature suggesting that physicians often receive limited formal education in capacity assessment, frequently report uncertainty regarding the practical and legal aspects of the evaluation process, and rely solely on subjective judgement rather than standardized, structured approaches [9]. This suggests that resident discomfort may extend beyond the assessment itself and include uncertainty regarding the implications of their findings and how to appropriately document them.

The educational intervention was intentionally designed around resident-identified needs. Previous research had been conducted after providing residents with an online self-directed module [10] and after standardized patient simulations [5]. However, instead of implementing a generalized curriculum, the pre-survey data guided development of a case-based didactic focused on Appelbaum’s criteria, indications for assessment, Tennessee’s state-specific legal considerations, and documentation strategies. Clinical tools including the Miller/Marin Algorithm [11], Aid to Capacity Evaluation [12], and Hopkins Competency Assessment Test [13] were also provided to residents. These instruments have previously been reviewed as validated methods that can improve the consistency and structure of capacity assessments when incorporated into clinical practice alongside physician judgment [14]. Following the intervention, statistically significant improvements were observed across all measured domains. The largest improvement was seen in overall knowledge, with a statistically significant increase of 2.61 points from 2.17 to 4.78 on a 5-point scale. Improvements were also statistically significant for all other measured domains, including documentation skills, understanding ethical versus legal considerations, capacity vs. competence, the four components of Appelbaum’s criteria, and confidence. Figure 1 demonstrates overall improvement for each of the six compared variables. 

Although direct comparison between studies is limited because of differing educational interventions and outcome measures, the consistent improvement observed across all assessed domains supports prior evidence that even brief, targeted educational experiences can substantially improve resident preparedness. The strong improvement in confidence is particularly important because clinicians who are uncomfortable assessing capacity may delay evaluations, rely excessively on consultants, or fail to recognize incapacity altogether. Improving physician confidence may therefore contribute to more timely assessments and more appropriate patient-centered decision-making. Raw data regarding survey responses that support the findings of this study are available from the corresponding author, upon request. 

Several limitations should be acknowledged. First, this study relied on self-reported measures of knowledge and confidence based on an unvalidated survey rather than objective assessments. Second, the post-survey response rate was lower than the pre-survey response rate, introducing the possibility of selection and response bias. Third, the study was conducted within residency programs at a single institution without a control group or pre- post-survey matching. This may limit generalizability to other training environments and validity. Finally, long-term retention of knowledge was not assessed, making it unclear whether improvements persist over time.

Future studies should evaluate objective measures of competency, including documentation quality and performance during simulated or observed capacity assessments. Previous reviews have similarly emphasized the need for empirically validated approaches to capacity assessment and continued investigation into methods that improve clinician performance and standardize evaluations [15]. Additionally, longitudinal follow-up could determine whether knowledge gains are maintained throughout residency training. Given the frequency with which residents encounter patients requiring capacity evaluations, incorporation of standardized capacity assessment curricula into graduate medical education may improve patient care, strengthen informed consent processes, and reduce missed cases of incapacity.

## Conclusions

This project demonstrates that resident physicians frequently assess capacity but often lack formal preparation, particularly in documentation and legal/ethical decision-making. A targeted needs assessment allowed the intervention to directly address resident-identified gaps, producing significant improvements in confidence and self-reported knowledge across all measured domains. These findings support incorporating structured capacity education into residency curricula, particularly in internal medicine and family medicine, where residents routinely care for acutely ill, cognitively impaired, psychiatric, intoxicated, or medically complex patients. Improved training may reduce missed incapacity, strengthen informed consent, improve documentation, and protect both patient autonomy and patient safety. Furthermore, this study demonstrates that a brief, needs-based educational intervention can produce meaningful improvements in resident preparedness, suggesting that capacity assessment education represents a high-yield addition to graduate medical education curricula.

## Appendices

Pre- and post-survey questions

https://drive.google.com/drive/folders/1RznQUQNDSlqWDO9M05T7dQXMMPlEeCW4?usp=sharing
